# Association between Serum Magnesium and Fractures: A Systematic Review and Meta-Analysis of Observational Studies

**DOI:** 10.3390/nu15061304

**Published:** 2023-03-07

**Authors:** Ligia J. Dominguez, Nicola Veronese, Stefano Ciriminna, José Luis Pérez-Albela, Vania Flora Vásquez-López, Santiago Rodas-Regalado, Giovanna Di Bella, Angela Parisi, Federica Tagliaferri, Mario Barbagallo

**Affiliations:** 1 Geriatric Unit, Department of Internal Medicine and Geriatrics, University of Palermo, 90133 Palermo, Italy; 2 Faculty of Medicine and Surgery, Kore University of Enna, 94100 Enna, Italy; 3 Instituto Bien de Salud, Lima 15036, Peru

**Keywords:** magnesium, fracture, osteoporosis, meta-analysis, risk factor, aging

## Abstract

Magnesium, an essential cation for numerous cellular processes, is a major component of bone. However, its relationship with the risk of fractures is still uncertain. The present systematic review and meta-analysis aim to investigate the impact of serum Mg on the risk of incident fractures. A systematic search was conducted using several databases including PubMed/Medline and Scopus from inception to 24 May 2022, including observational studies investigating serum Mg and the incidence of fractures considered as outcomes. Abstract and full-text screenings, data extractions, and risk of bias assessments were conducted by two investigators independently. Any inconsistencies were resolved by consensus with a third author. The Newcastle–Ottawa Scale was used to assess the study quality/risk of bias. Among 1332 records initially screened, 16 were retrieved as full-texts; of them, four papers were included in the systematic review with a total of 119,755 participants. We found that lower serum Mg concentrations were associated with a significantly higher risk of incident fractures (RR = 1.579; 95%CI: 1.216–2.051; *p* = 0.001; I^2^ = 46.9%). Our systematic review with meta-analysis suggests a strong association of serum Mg concentrations with incident fractures. Further research is needed to confirm our results among other populations and to assess whether serum Mg is potentially relevant in the prevention of fractures, which continue to increase and represent a significant health burden due to the associated disability.

## 1. Introduction

Fragility fractures are unquestionably a common relevant public health problem in terms of patient’s health, quality of life, as well as social and financial burden [1,2,3]. The incidence of fragility fractures increases strikingly with age [3,4,5], which makes them continue to rise due to the aging of the population worldwide [6]. The lifetime risk of major osteoporotic fractures at the age of 50 years is 22% in men and 46% in women. It is estimated that over one-third of all world osteoporotic fractures occurred in Europe in 2000 [2,3,7]. 

Fragility fractures not only entail direct costs but also expenses deriving from the disability they cause. The disability adjusted life years (DALYs) attributed to fragility fractures are two million every year in Europe, which exceeds those attributable to hypertensive heart disease [1,4]. There is evidence supporting that screening for high fracture risk in primary care ought to be considered for inclusion into health care systems to lessen the burden of fractures, particularly hip fractures [3]. 

The mineral components of the body are factors accepted as key determinants of bone health. This is another relevant target for prevention given its potential modifiable nature. Evidence in this regard has focused mainly on calcium and vitamin D despite the fact that there are many other minerals and dietary components that may also contribute [8]. There is evidence from experimental studies showing that magnesium (Mg) deficit is associated with modifications in osteoclasts and osteoblasts number and activity [9,10,11,12]. As such, former investigations by Rude et al. found that osteoclast numbers were significantly increased with Mg depletion [9]. Additionally, several studies have demonstrated in experimental conditions that lower Mg is associated with increased osteoclast activity and decreased osteoblast activity, suggesting that that this effect could be reversed by Mg supplementation [10,11,13,14,15]. 

Mg, a crucial element for a number of cellular processes, including over 600 enzymatic reactions, all oxidative phosphorylation processes, protein synthesis, energy production reactions, nucleic acid synthesis and stability, as well as glycolysis [16], is also a major component of bone. About 67 percent of body Mg is found in bone tissue [17].

Even if the Mg cation has the biological plausibility to be a key element in bone health, the body status of Mg in relation to the incidence of fractures is until uncertain today. The studies found currently in the medical literature mainly refer to its intake with diet, with results showing that Mg is associated with an increased bone mineral density [18,19,20,21] and a tendency to lower incident fractures [20,22]. For the improvement in bone density there is more solid evidence than for incident fractures. For this latter outcome, most of the few available studies are small or poorly designed, not allowing us to formulate definitive conclusions [20].

There are also studies with discordant results and not always optimal designs that evaluate the association of serum Mg with the incidence of fractures. Therefore, the aim of the present systematic review and meta-analysis was to investigate the impact of serum Mg on fracture risk, including observational studies dealing with this specific problem.

## 2. Materials and Methods

This systematic review adhered to the PRISMA statement [23] and followed a pre-planned but unpublished protocol. The adherence to the PRISMA 2020 checklist is reported as Supplementary Material. 

### 2.1. Data Sources and Searches

Two investigators (NV and SC) independently conducted a literature search using several databases, including PubMed/Medline and Scopus, from database inception to 24 May 2022, involving observational studies investigating serum Mg and the incidence of fractures considered as outcomes. 

The search strategy included the concepts of Mg and fractures (magnes* AND fractur* in Pubmed) and adapted for Scopus. Any inconsistencies were resolved by consensus with a third author (LJD). No automation tool was used.

### 2.2. Study Selection

For the inclusion of studies in the meta-analysis, we considered the following criteria: (i) observational studies (case-control and longitudinal); (ii) evaluation of Mg in serum at the baseline evaluation; (iii) reporting data regarding incident osteoporotic fractures; and (iv) written in English. Studies were excluded if they: (i) did not include humans; (ii) assessed Mg from other sources (e.g., dietary); or (iii) were cross-sectional. The studies were grouped according to the estimates reported. Two reviewers (SC and NV) independently screened the studies, and a third senior investigator (LJD) was available in case of divergency. 

### 2.3. Data Extraction

Two investigators (NV and SC) independently extracted the key data from each included article by means of a standardized Excel spreadsheet. A third investigator (LJD) independently revised the data. Data extracted from each article comprised: author names, year of publication, country, condition, study design, main condition, demographic information, follow-up duration (in months), type of fractures, number and type of confounders used in multivariate analyses. 

### 2.4. Outcomes

The primary outcome was the incidence of any osteoporotic fracture, total and by site (hip, vertebral, wrist, others). All the outcomes were compatible each other. 

### 2.5. Quality Assessment

For assessing the study quality/risk of bias, we used the Newcastle–Ottawa Scale (NOS) [24]. The NOS assigns a maximum of nine points based on three quality parameters: selection, comparability, and outcome. The initial evaluation was independently conducted by one of the authors (NV) and afterwards revised by another independent author (SC). In the NOS, the risk of bias was then considered as high (<5/9 points), moderate (6–7/9), or low (8–9/9) [25]. No automation tool was used. 

### 2.6. Data Synthesis and Analysis

All analyses were performed using STATA version 14.0 (StataCorp). The primary analysis investigated the incidence of fractures by serum Mg levels at the baseline. We calculated the risk ratios (RRs), adjusted for the highest number of potential confounders available in each analysis with their 95% confidence intervals (CIs), applying a random-effect model [26]. For all the analyses, we considered the highest quantile, representing a better Mg status, as reference. Heterogeneity across studies was assessed by the I^2^ metric and χ^2^ statistics. Given the significant heterogeneity (I^2^ ≥ 50%, *p* < 0.05), and for outcomes having at least ten studies, we plan to conduct a series of meta-regression analyses, according to follow-up, serum Mg levels, mean age and percentage of women. 

Publication bias was assessed by visually inspecting funnel plots and using the Eger bias test [27]. The trim-and-fill analysis was used for addressing this issue [28].

For all analyses, a *p*-value less than 0.05 was considered statistically significant.

## 3. Results

### 3.1. Search Results

**Figure 1:**
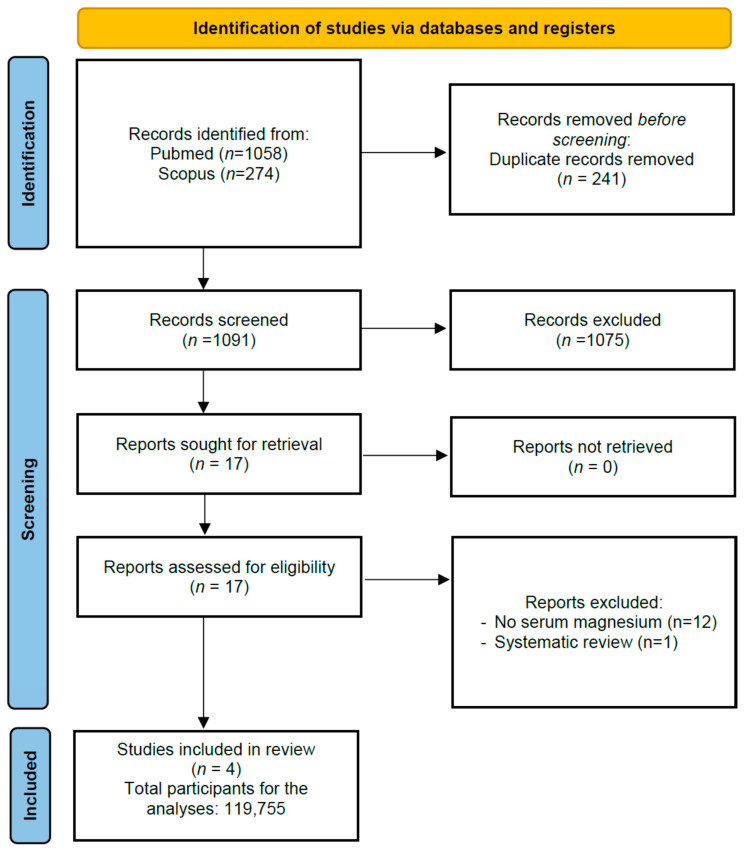
PRISMA flow-chart.

Figure 1 shows the PRISMA flow-chart. Among 1332 records that were initially screened, 17 were retrieved as full texts. After further exclusions (12 without serum Mg and 1 corresponding to a systematic review), four papers [29,30,31,32] were eligible for inclusion in the systematic review. 

### 3.2. Study and Patient Characteristics; Meta-Analysis

Table 1 reports the full details regarding the descriptive findings of the four eligible studies. Overall, two studies were performed in Japan and the other two in Europe. Three studies had a longitudinal, prospective cohort design, and the other one was retrospective. Two studies were carried out among patients undergoing hemodialysis and two in the general population.

A total of 119,755 participants were followed up for a mean of 79 months (range: 24 to 161). The mean age was 62 years, with a mean percentage of 33% women. The analyses were adjusted for a mean of 15 potential confounders.

**Figure 2:**
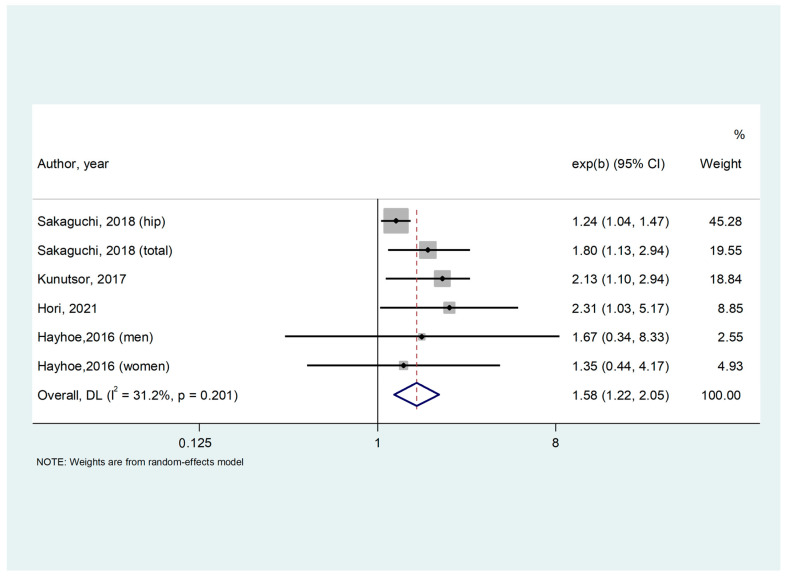
Forrest plot of association between low serum magnesium and incident fractures [29,30,31,32].

As reported in Figure 2, lower serum Mg concentrations were associated with a significantly higher risk of incident fractures (RR = 1.579; 95% CI: 1.216–2.051; *p* = 0.001; I^2^ = 46.9%). The results were not affected by any heterogeneity (I^2^ = 31.2%, *p* = 0.201) nor publication bias (Egger’s test = 0.94 ± 0.43; *p* = 0.10). After trimming, nine studies were to the left of the mean, and the recalculated effect size was only slightly reduced (RR = 1.25; 95% CI: 1.09–1.43).

### 3.3. Risk of Bias

The Newcastle–Ottawa Scale did not indicate any possible risk of bias since, in all the studies included, the outcomes were well-described and according to validated criteria, the follow-up was long enough and for detecting future fractures, and the follow-up was adequate for detecting the outcomes of interest, i.e., fractures.

## 4. Discussion

In the present systematic review and meta-analysis, we aimed to examine the impact of serum Mg on the risk of incident fracture in the available suitable studies. Starting from over 1300 studies of the original search and after reviewing them according to the latest PRISMA recommendations, four studies with a total of 119,755 participants were available and were included in the analyses. All the four studies included in the meta-analysis were of high quality (Newcastle–Ottawa Scale of 9 for all). We observed a strong relationship of a lower serum Mg concentration with a higher risk of incident fractures. These results confirm the key role that Mg may play on bone health and entail an open opportunity that can help in fracture prevention since hypomagnesemia is a potentially modifiable factor.

The number of quality studies specifically investigating the relationship between serum Mg concentrations and the risk of fractures is reduced to a few. The four studies included in the meta-analysis after the PRISMA selection procedure were from Japan and Europe [29,30,31,32]. Hayhoe et al. conducted a study in a UK random subset of 4000 participants from the European Prospective Investigation into Cancer and Nutrition (EPIC)-Norfolk cohort of 25,639 men and women. Multivariate adjusted regression analyses were performed to investigate the associations of fractures with serum Mg concentration. The authors reported statistically significant trends in fracture risk in men across serum Mg concentration for spine fractures (*p* = 0.02) and total hip, spine, and wrist fractures (*p* = 0.02). None of these individual statistically significant associations remained after an adjustment for multiple testing [29].

The second study included in the present meta-analysis evaluated the association of baseline serum Mg concentrations with the risk of incident fractures in 2245 men (aged 42 to 61 years) from the Kuopio Ischemic Heart Disease prospective cohort study. After a median follow-up of 25.6 years, serum Mg was non-linearly associated with the incidence of total fractures. The age-adjusted hazard ratio (HR) (95% CIs) for total fracture risk for participants at the bottom quartile vs. those at the top quartile of serum Mg was 2.10 (1.30–3.41), which persisted after adjustment for multiple confounders (1.99; 1.23–3.24). Even after further adjustments for relevant parameters, such as renal function, socioeconomic status, total energy, and several trace elements, the HR remained statistically significant: 1.80 (1.10–2.94). Likewise, for femoral fractures, the HR [CIs] was also significant (2.13 [1.13–3.99]) in the fully adjusted analysis. These results strongly confirm the possibility of intervening in low Mg status to prevent fractures in Caucasian men [30].

The third study used a nationwide database of patients undergoing dialysis in Japan. The authors identified 113,683 patients on dialysis treatment without a history of hip fracture at baseline. Multivariate logistic regression analyses showed that patients in the lower quartile of serum Mg had a 1.23-fold higher risk of hip fracture compared to those in the highest quartile (95% CIs: 1.06–1.44; *p* < 0.01), with a dose-response relationship between serum Mg quartile and incident hip fracture risk. These results were consistent for both men and women. Considering serum Mg concentration as a continuous variable, each 1-mg/dL increase in serum Mg was associated with a 14.3% decreased risk in the incidence of hip fracture (95% CI: 3.8 to 23.8; *p* < 0.01). The fraction of serum Mg concentration attributable to the population for incident hip fractures was 13.7% (95% CI: 3.7% to 22.7%), which was considerably higher than that of serum calcium, serum phosphate, and parathyroid hormone (PTH) levels [31].

The fourth study included in our meta-analysis was a longitudinal study conducted among 358 patients undergoing hemodialysis treatment. The cumulative incidence rates of fractures were strikingly different in participants with lower serum Mg (below 2.6 mg/dL) vs. those with higher Mg circulating concentrations: 17.6% vs. 5.2% with a multi-adjusted HR of 2.31 (95% CI of 1.03, 5.17; *p* = 0.03). Participants with lower Mg and lower BMD had a 9.21-fold higher risk of fractures (95% CI of 2.35–47.0; *p* < 0.001) when compared to participants with higher serum Mg concentrations and high BMD. Therefore, adding Mg levels and lumbar spine BMD resulted in a significant improvement of the prediction of fractures [32].

Considering that two of the studies suitable for inclusion in our meta-analysis were carried out in patients undergoing dialysis, it is worth mentioning that lately more attention has been paid to the important role played by the kidney in Mg homeostasis and of Mg in the comorbidities associated with chronic kidney disease (CKD). For example, the increased all-cause and cardiovascular mortality risk of patients with CKD persist even after adjustments for traditional cardiovascular risk factors, indicating that other specific factors for the kidney may contribute to this risk [33]. Recently, low serum Mg concentrations have been suggested to be one of these factors [34]. Increasing dietary Mg in experimental models of CKD decreased the development of arterial calcification [35], a well-known complication of CKD. It is widely accepted that arterial calcification is an important pathophysiological process leading to CVD in patients with CKD and a strong predictor of mortality [36,37,38]. About 80 to 90 percent of patients with end-stage renal disease (ESRD) have vascular calcifications [39]. Coronary artery calcifications are independently and significantly associated with the risk of CVD, myocardial infarction, and heart failure in patients with CKD [40].

Bone metabolism disorders and the tendency to develop vascular calcifications in CKD patients seem to be deeply connected. Reduced bone formation has been associated with coronary calcifications in CKD patients not yet on dialysis [41]. Some studies suggest a link between low bone density and arterial calcifications and vascular stiffness in patients with ESDR [42,43]. It has been reported that Mg prevents calcifications in cultured smooth muscle vascular cells and in aortic tissue through the inhibition of enzymes that contribute to cardiac remodeling [44,45]. In patients with CKD, Mg has shown beneficial effects on multiple surrogate outcome parameters of CVD related to vascular calcifications, such as calcification propensity score and carotid intima media thickness [46,47,48].

There is consistent evidence suggesting that Mg has beneficial effects on bone health. Yet, most of the available studies refer to dietary Mg as an exposure and very few to serum Mg concentrations. In this regard, there is convincing evidence that dietary Mg is associated with increased BMD, while the evidence is less homogeneous when it comes to fractures [18,21,49].

Several mechanisms may help to explain our findings of a strong association between serum Mg and incident fractures. This includes the modulation of osteoclastic and osteoblastic activity demonstrated for Mg [12,13,14,15,50,51]; the significant effects of Mg on bone density both in experimental models [11] and in clinical studies [18,19,20,21,52]; the alterations of other key nutrient necessary for bone health, such as calcium, as well as the importance of optimal levels of Mg for the proper functioning of vitamin/hormone D [53,54,55,56,57] and PTH [58,59,60,61,62,63,64], crucial modulators of bone health; and the association of low Mg status with inflammation [65,66,67,68], which has been associated with poor bone quality and skeletal fragility [69].

Regarding the modulation of osteoblastic and osteoclastic activity, in addition to the initial investigations by Rude et al. showing an increased number of osteoclasts in experimental animals receiving an Mg-deficient diet [9], there are recent interesting reports on the association of low Mg with increased osteoclastic activity and a reduction in osteoblastic activity [13,14,15]. For example, an experimental study in mice evaluated the effect of Mg deficiency on osteoclast precursor viability and proliferation, as well as on the mRNA expression of genes related to osteoclastogenesis and genes related to Mg. Mg deficiency resulted in increased numbers of osteoclast-like cells from different bones (long and jaw bone) and a higher expression of genes related to osteoclasts [13].

Some of these recent studies have been carried out in experiments seeking to develop and improve Mg-based bone implants, an area of recent and growing interest due to the fact that Mg is both biocompatible with and biodegradable in the human body and stimulates bio-responses at cellular and molecular levels, with the potential promotion of bone remodeling [70]. A study examining the role of Mg ions in the migration of osteoblast-like cell lines found that Mg treatment increased the protein expression level of an epithelial-mesenchymal transition marker. In addition, the junctional site localization of Zona-occludens 1, a representative tight junction protein, was destroyed by Mg treatment, its cytoplasmic localization increased, and alkaline phosphatase activity also increased. These results denoting the mechanism by which Mg is involved in osteoblast migration may be relevant for fracture healing, potentially contributing to help understand the bone-formation process in patients with osteoporosis and musculoskeletal injury [14].

Another study explored whether the coculture of osteoblasts and osteoclast in Mg-based implants may influence bone metabolism and remodeling upon degradation. In this study by Wu et al., cocultures containing both bone-forming osteoblasts and bone-resorbing osteoclasts significantly enhanced the formation of osteoblasts, whereas they decreased osteoclastogenesis in the cultures with high concentrations of Mg extract [15].

Thus, Mg-based alloys have become a relevant category of materials that is attracting more and more attention due to their high potential use as orthopedic temporary implants, being a viable alternative to non-degradable metals implants. Generally, temporary orthopedic implants need to be removed from the body after a certain period. Conversely, biodegradable implants, such as Mg-based implants, eliminate the requirement of a second surgery to remove the planted implants [70].

Another key mechanism that may help explain our findings is the relationship between vitamin D and Mg. The effects of vitamin D as a key regulator of calcium and phosphate metabolism is well known [71]. It is key to remember that several stages in the metabolism of vitamin/hormone D, including the hepatic synthesis of 25-hydroxyvitamin D (25OHD) and binding to its transport protein, as well as the activation of vitamin D into 1,25[OH]_2_D, the active form of the hormone by renal hydroxylation, have Mg as a cofactor [53,54,55,56,57]. Consequently, in the presence of Mg deficit, vitamin D’s actions may be reduced. In addition, Mg plays a crucial role in the synthesis and metabolism of PTH. Thus, Mg deficit impedes PTH secretion and/or synthesis [58,59,60,61]. Patients with Mg deficiency may present hypocalcemia in spite of high PTH levels, suggesting resistance to PTH action in bone and kidney [62]. Hypocalcemia due to Mg deficit, secondary to decreased PTH secretion or peripheral PTH resistance, is additionally deteriorated by a lack of the PTH stimulation of renal 1-alpha-hydroxylation, with the further worsening of vitamin D deficit [53]. These events, that is, Mg deficiency that leads to reduced 1,25(OH)_2_D synthesis and impaired PTH response, have been implicated in the described condition “Mg-dependent vitamin D-resistant rickets” [54,63]. It has been formerly shown that an infusion of Mg stimulated a non-significant increase in 1,25(OH)_2_D and 25OHD [53], whereas an infusion of Mg infusion plus vitamin D p.o. significantly increased both 1,25(OH)_2_D and 25OHD [64], confirming the interaction between Mg and vitamin D. It is worth mentioning the fact that vitamin D plays a key role not only in calcium metabolism but also in Mg metabolism through the stimulation of intestinal Mg absorption and prevention of renal Mg excretion [72]. Thus, it seems that the deficit of both compounds, Mg and vitamin D, so prevalent today, feed each other, which may further worsen both deficits. The presence of Mg and vitamin D deficiency concurrently may cause clinically significant outcomes, including an increased risk of fragility fractures [73].

Another of the key mechanisms that can help explain our results is related to the antioxidant and anti-inflammatory properties of Mg [65,66,67,68,74]. Indeed, inflammation and oxidative stress are accepted mechanisms mediating bone fragility [69]. A number of experimental studies have shown that Mg deficiency causes the elevation of proinflammatory molecules IL-6, TNF-alfa, VCAM-1, IL-1-beta, and PAI-1 [66,67]; increased circulating inflammatory cells [68]; and the augmented production and release of acute phase proteins (i.e., complement, fibrinogen, and alfa2-macroglobulin) in the liver [65,66].

Clinical studies have demonstrated that low serum Mg concentrations as well as diets poor in Mg are robustly related to low-grade systemic inflammation [75,76,77]. Other studies have reported an inverse relationship of inflammation markers with dietary Mg intake and serum Mg. The Women’s Health Study reported that Mg dietary intake was inversely associated with serum CRP concentrations [78]. Likewise, analyses of data from the 1999–2002 NHANES databases showed that Mg intake was inversely associated with CRP concentrations. Among 70% of the population studied, who were not taking Mg supplements, dietary Mg intake lower than the RDA was significantly associated with an increased risk of an elevated CRP [76]. A large study from Finland recently confirmed the significantly inverse relationship of low dietary Mg intake with serum hs-CRP concentrations [79]. A recent systematic review and meta-analysis of 17 RCTs (889 participants) investigating the effects of Mg supplements vs. placebo on serum markers of inflammation reported a significantly decreased serum CRP and increased nitric oxide levels with Mg supplementation vs. placebo. Mg supplementation also significantly reduced other inflammatory markers, including plasma fibrinogen, tumor necrosis factor-ligand superfamily member 13B, ST2 protein, tartrate-resistant acid phosphatase type 5, and IL-1 [74] confirming the robust anti-inflammatory actions of Mg.

Low serum Mg concentrations are primarily due to a poor Mg diet, a prevalent feature of the Western diet. According to the Dietary Guidelines for Americans, about 49% of the USA population (comprising all age groups), had an Mg intake below the estimated average requirement [80]. There are other estimates showing that more than 60% of Americans do not meet the recommendation due to low Mg daily intake [76]. This is most likely due to the eating habits of the population with diets in which the main components are processed and ultra-processed products, with this industrial processing being responsible for the loss of Mg in this type of food [81].

The findings of the present meta-analysis should be interpreted with its limitations. First, only four studies were identified as suitable for the analyses according to PRISMA recommendations. Although it is a small number, these studies were of good quality and the number of participants was adequate (over 100,000). Another limitation is that the studies were conducted in Japan and Europe and two of the studies included hemodialysis patients. Future studies conducted in other populations (e.g., American and other Asian countries) are needed to test whether our results are replicated and extendable to those populations. The other limitation concerns serum Mg, a method used to evaluate Mg status in daily clinical practice. Mg is mainly an intracellular ion, with only 1% of total body Mg contained in the blood. Thus, serum Mg may not accurately reflect the global body Mg status because it drops only when the tissue deposits are exhausted; therefore, it is unable to disclose most of the mild to moderate Mg deficits, which are often underestimated and unrecognized. Furthermore, subclinical Mg deficit symptoms are generally not specific and are not easily connected to unobserved electrolyte alteration by the clinician [16,82]. Nevertheless, serum Mg is the parameter currently available in the studies. It is expected that in the future a practical method for measuring intracellular Mg will be developed that can be used routinely in the clinical setting.

## 5. Conclusions

Our systematic review with meta-analysis suggests a strong association of serum Mg concentrations with the risk of incident fractures. Further research is needed to replicate the present results in other populations as well as to assess the potential significance of serum Mg in the prevention of fractures, which continue to increase and represent a significant health burden due to the associated disability.

## Figures and Tables

**Table 1:** Descriptive findings of the studies included.

Author, Year	Country	Study Design	Sample Size	Condition	Mean Age (SD)	Females (%)	Mean Follow-Up (Months)	Type of Fractures	Confounders	Newcastle–Ottawa Scale
Hayhoe, 2016 [29]	Multicentric Europe	Population-based Prospective Cohort	3469	General population	62.5 (9.3)	57.9	161	Total	Age, BMI, smoking status, physical activity, family history of osteoporosis, menopausal and HRT status in women, corticosteroid use.	9
Kunutsor, 2017 [30]	Finland	Population-based Prospective Cohort	2245	General population	53.1 (5)	0	69	Hip	Age, BMI, height, systolic BP, smoking, history of diabetes, alcohol consumption, physical activity, estimated GFR, SS, total energy intake, serum zinc, serum potassium, and serum ionized calcium	9
Sakaguchi, 2018 [31]	Japan	Observational, Cohort Study	113,683	Hemodyalisis	64.9 (12.3)	37.5	24	Hip and total	Age, sex, BMI, dialysis duration, urea reduction rate, dialysis vintage, physical activity, DM, Ca, P, ALP, intact PTH, albumin, CRP, hemoglobin, past history of CVD (MI, cerebral infarction, cerebral hemorrhage, and amputation), medication (Ca carbonate, sevelamer hydrochloride, lanthanum carbonate, active vitamin D analog [i.v. and p.o.], and cinacalcet hydrochloride), history of parathyroidectomy	9
Hori, 2021 [32]	Japan	Retrospective	358	Hemodyalisis	65.6 (14.3)	36	36	Total	Age, BMI, HD duration, past incident fracture, use of phosphate binders, total hip BMD	9

ALP: alkaline phosphatase; BMD: bone mineral density; BMI: body mass index; BP: blood pressure; Ca: calcium; CVD: cardiovascular disease; CRP: C-reactive protein; DM: diabetes mellitus; GFR: glomerual filtration rate; HD: hemodialysis; i.v.: intravenous; P: phosphate; p.o.: oral; PTH: parathyroid hormone; SD: standard deviation; SS: socioeconomic status.

## Data Availability

The data and the databases are available upon reasonable request to the Corresponding Author.

## Supplementary Materials

The following supporting information can be downloaded at: https://www.mdpi.com/article/10.3390/nu15061304/s1. PRISMA 2020 Checklis.
